# Hospitalization budget impact during the COVID-19 pandemic in Spain

**DOI:** 10.1186/s13561-021-00340-0

**Published:** 2021-11-03

**Authors:** F. J. Carrera-Hueso, L. Álvarez-Arroyo, J. E. Poquet-Jornet, P. Vázquez-Ferreiro, R. Martínez-Gonzalbez, D. El-Qutob, M. A. Ramón-Barrios, F. Martínez-Martínez, J. L. Poveda-Andrés, C. Crespo-Palomo

**Affiliations:** 1 Pharmacy Service, University Hospital La Plana, Carretera de Vila-real a Burriana, Km. 0.5, 12540 Villarreal, Castellón, Spain; 2 Pharmacy Doctoral Program at University of Granada, Granada, Spain; 3 Pharmacy Service, Hospital de Denia (Marina Salud), Denia (Alicante), Spain; 4 Ophtalmology Department, Hospital Virxe da Xunqueira, Cee (A Coruña), Spain; 5 Informatics and computer Service, University Hospital La Plana, Villarreal (Castelló), Spain; 6 Allergy Service, University Hospital La Plana, Villarreal (Castelló), Spain; 7 Internal Medicine Unit, University Hospital Doctor Peset, Valencia, Spain; 8 Grupo Investigación de Atención Farmacéutica, Pharmacy and Pharmaceutical Technology Department, University of Granada, Granada, Spain; 9 Pharmacy Department, Hospital Universitari i Politecnic La Fe, Valencia, Spain; 10 Department G.M. statistics, University of Barcelona, Barcelona, Spain; 11 Axentiva Solutions, Barcelona, Spain

**Keywords:** Costs and cost analysis, COVID-19, Health care costs, Clinical laboratory tests, Hospitalization, Budgets

## Abstract

**Objectives:**

The aim was to determine the direct impact of the COVID-19 pandemic on Spain’s health budget.

**Methods:**

Budget impact analyses based on retrospective data from patients with suspected severe acute respiratory syndrome coronavirus 2 (SARS-CoV-2) admitted to a Spanish hospital between February 26 and May 21, 2020. Direct medical costs from the perspective of the hospital were calculated. We analyzed diagnostic tests, drugs, medical and nursing care, and isolation ward and ICU stays for three cohorts: patients seen in the emergency room only, hospitalized patients who tested positive for SARS-CoV-2, and patients who tested negative.

**Results:**

The impact on the hospital’s budget for the 3 months was calculated at €15,633,180, 97.4% of which was related to health care and hospitalization. ICU stays accounted for 5.3% of the total costs. The mean cost per patient was €10,744. The main costs were staffing costs (10,131 to 11,357 €/patient for physicians and 10,274 to 11,215 €/patient for nurses). Scenario analysis showed that the range of hospital expenditure was between €14,693,256 and €16,524,924. The median impact of the pandemic on the Spanish health budget in the sensitivity analysis using bootstrapped individual data was €9357 million (interquartile range [IQR], 9071 to 9689) for the conservative scenario (113,588 hospital admissions and 11,664 ICU admissions) and €10,385 million (IQR, 110,030 to 10,758) for the worst-case scenario (including suspected cases).

**Conclusion:**

The impact of COVID-19 on the Spanish public health budget (12.3% of total public health expenditure) is greater than multiple sclerosis, cancer and diabetes cost.

**Supplementary Information:**

The online version contains supplementary material available at 10.1186/s13561-021-00340-0.

## Introduction

Coronavirus disease 2019 (COVID-19), caused by severe acute respiratory syndrome coronavirus 2 (SARS-CoV-2), was declared a pandemic by the World Health Organization. By the end of June, it had affected 188 countries, with over 9 million confirmed cases and rising infection rates [1]. A variety of public health measures have been adopted to control the pandemic and ease the burden on healthcare systems [2].

The long-term health consequences and potential sequelae of COVID-19 are unknown [3, 4], but the social and economic impacts are already worse than those of the Second World War [3]. While major COVID-19-related research efforts are underway, there is a paucity of studies examining the impacts of the pandemic on public health budgets. This information is crucial to correctly manage the ongoing crisis, prepare for second waves [5], and guide the implementation and management of new services such as telemedicine or the creation of dedicated COVID-19 clinic units. Budget information is also needed to establish intervention-specific costs and is essential for analyzing the cost-effectiveness of new treatments for COVID-19. It can also help identify diagnostic groups that should allow for improved management.

The aim of this study was to conduct a hospital budget impact analysis to assess the impact of COVID-19 at its peak on the Spanish public health budget.

## Methods

### Study design, population, and resources

We estimated the impact of COVID-19, considering all direct medical costs, on the Spanish public health budget by extrapolating data from a retrospective cohort study covering a period of 86 days during the peak of the pandemic. The study was conducted in a 252-bed Spanish hospital that serves a catchment area of 187,258 people. It was registered with the Spanish Agency of Medicines and Medical Products (AEMPS) and approved by the hospital’s ethics committee. Verbal informed consent in the presence of a witness was obtained from all patients and noted in the patients’ medical records. This procedure was authorized by the Spanish Health authorities in view of the exceptional epidemiological situation. Eligible patients were adults (> 18 years) with suspected COVID-19 who visited the hospital’s emergency room (ER) between February 26 and May 21, 2020. Pregnant women were excluded. In cases of readmission, data from the first admission only were analyzed.

The patients were classified into three cohorts: 1) those not requiring hospital admission (ER cohort), 2) those admitted to the hospital and who received a positive real-time polymerase chain reaction (RT-PCR) result within 24 h (SARS-CoV-2-positive cohort), and 3) those admitted to the hospital and who received a negative RT-PCR result within 24 h (SARS-CoV-2-negative cohort).

Individual records of all patients in the ER cohort were reviewed to identify the tests performed (RT-PCR, chest X-ray, and blood tests [complete blood count, biochemical parameters, coagulation test]. A record was also made of time spent in the ER for all patients. Stays of 16 h or less were classified as medical visits, while longer stays were classified as ER stays.

Individual information was also collected on all tests performed during hospitalization. These included RT-PCR, imaging studies, blood tests [complete blood count, biochemical parameters, coagulation test], blood gas analysis, other laboratory tests (ferritin, D-dimer, C-reactive protein, and procalcitonin), and microbiological tests (blood cultures and testing for multiple atypical respiratory pathogens). We also calculated lengths of intensive care unit stays (ICU stays) and isolation ward stays (general stays). For the SARS-CoV-2-negative cohort, we calculated resources used in the first 48 h of hospitalization, as this was the maximum time for receiving RT-PCR results. We also analyzed survival at the end of follow-up for SARS-CoV-2-positive and -negative cohorts.

### Costs

Direct medical costs from the perspective of the hospital were calculated in 2020 Euros (€). Discounts and indirect or intangible costs were not considered. Unit costs for ER and ICU stays, hospitalization, and staff salaries were obtained from the official rates established for our hospital for 2020 and checked against rates for several hospitals in different regions of Spain [6]. These were then multiplied by resource use data for each cohort to provide a combined total. Drug prices were obtained from the hospital’s pharmacy department. The main drug groups considered were antivirals, anti-inflammatories, antibiotics, antihypertensives, and gastroprotectives. We also computed the costs of laboratory tests and imaging studies performed during hospitalization. We do not include in our analyses indirect cost because our cohort is mainly hospitalized patients.

Once we had calculated the costs for the three cohorts, we estimated the impact of the COVID-19 pandemic for the period analyzed on the hospital’s budget for each cohort. The patients in the SARS-CoV-2-negative cohort were included as they had come to the hospital because they thought they had COVID-19 and would not have come had the pandemic not existed. As these criteria might vary according to the research team, we applied two additional approaches to estimate the total impact.

1. In the first case, we calculated the impact of the pandemic for ER and SARS-CoV-2-positive cohorts only.
2. In the second case, we estimated a false-negative rate of 29% [7], as the true rate was not available. In other words, we assumed that 29% of SARS-CoV-2-negative patients were actually infected and would have returned to the hospital for care, thereby adding to the costs.

### Estimation of the National Level

Using official data reported for COVID-19-related hospital and ICU stays in Spain in June 2020 [8], we estimated the global impact of COVID-19 on the Spanish health budget using a linear approach. The number of cases for the ER cost analysis was estimated by calculating the ratio of SARS-CoV-2-positive patients to both ER patients (1:3.58) and SARS-CoV-2-negative patients (1:1.32) at our hospital.

We evaluated two scenarios: a conservative scenario for which we calculated the costs associated with the official cases reported for Spanish hospitals (113,558 hospital admissions and 11,664 ICU admissions) [8] and a worst-case scenario for which we assumed that 52.2% of SARS-CoV-2-positive patients would be hospitalized and that 5.9% of these would require ICU admission [8]. In these cases, the missing values were filled using linear interpolation, giving 115,877 hospital admissions and 14,806 ICU admissions.

### Sensitivity analyses

We performed univariate sensitivity analysis to assess the uncertainty surrounding all the parameters in our study. Considering the potential uncertainty arising from differences in clinical practice across Spain, we estimated and compared ranges of unit costs for seven autonomous communities: Andalusia, the Canary Islands, Cantabria, Catalonia, Madrid, Navarre, and the Basque Country (supplementary material Table S1).

We finally performed a probabilistic sensitivity analysis by bootstrapping individual patient data to obtain the most realistic estimates possible [9]. The bootstrap approach is a non-parametric method that makes no distributional assumptions concerning the statistic in question. Instead, it employs the original data in a resampling exercise in order to give an empirical estimate of the sampling distribution of that estimate keeping the correlations between the costs and effects of our population.

We generated 1500 bootstrap samples for each cohort using the size of the original sample and performing resampling with replacement. For each subsample, we calculated mean costs and budget impacts for the reference hospital and Spain as a whole.

### Main assumptions

1. Personal protective equipment (PPE) costs (which have spiraled during the COVID-19 pandemic) were calculated as hospitalization costs. Indirect costs were not included for the lack of information.
2. Even though PPE and disinfectant costs have increased substantially because of the pandemic, price increases were not contemplated in our analyses.
3. Staff overtime telework outside ordinary working hours, and increased workload in other departments (e.g., laundry services) were not considered.
4. Future cost projections were not calculated, as the COVID-19 sequelae are not yet well known. Individual patient requirements during hospitalization or after discharge were also not contemplated.
5. Even though antibiotics are not recommended as prophylactic agents for COVID-19, they were included in the cost analyses as they are part of the workflow at our hospital.
6. The calculations for ICU costs included unit costs and complete blood count, blood gas analysis, and chest X-ray costs.

Microsoft Excel 2010 was used for analyzing the initial scenario, modelling, bootstrapping, and the sensitivity analysis, while SPSS for Windows 26 (IBM Corp. Released 2010) was used to compare cohorts.

## Results

We evaluated 1602 patients, of whom 1446 fulfilled the inclusion criteria. Their characteristics are summarized in Table 1. Between February 26 and May 21, 2020, 912 patients tested positive for SARS-CoV-2 by RT-PCR. Based on the total catchment population for our hospital (187,258), this corresponds to a rate of 487 cases per 100,000 population.

**Table 1:** General characteristics of the study population

	ER cohort	SARS-CoV-2-positive cohort	SARS-CoV-2-negative cohort
Total patients, n	989	265	348
< 18 years old, n	111	2	7
Readmissions, n	–	9	18
Patients included, N	878	254	323
Age, mean (SD)	49.3 (16.2)	68.4 (15.9)	70.7 (17.9)
Male, n (%)	381 (43.4)	139 (54.7)	174 (53.9)
Deaths, n (%)	3 (0.3)	43 (16.9)	32 (9.9)
Days of hospitalization, mean (SD)	1 (0.1) *	44.1 (4.8)	8.3 (1.7)
Days in ICU, mean (SD)	0 (0.1)	37.8 (4.3)	4.7 (1.9)

ER, emergency room; ICU, intensive care unit; SARS-CoV-2, severe acute respiratory syndrome coronavirus 2; SD, standard deviation; ICU, intensive care unit

^*^ Stays of 16 h or less were classified as medical visits, while longer stays were classified as ER stays

The total estimated impact of COVID-19 on the hospital’s budget for the 86 days analyzed was €15,633,180. The vast bulk of this spending (94.7%) was related to the treatment and management of SARS-CoV-2-positive patients admitted to hospital. The mean cost per patient was €10,744 (€307 for the ER cohort, €1710 for the SARS-CoV-2-negative cohort, €50,132 for the SARS-CoV-2-positive cohort without ICU admission, and €280,956 for the SARS-CoV-2 positive cohort with ICU admission. The breakdown of costs per cohort is given in Table 2.

**Table 2:** Impact on study hospital budget

Cost in € 2020			
Items	ER cohort*	SARS-CoV-2-positive cohort	SARS-CoV-2-negative cohort
***Diagnostic tests***			
RT-PCR for SARS-CoV-2	47,855.80	25,673.99	20,888,41
Complete blood count	–	3745.74	1949,48
Laboratory test†	19,075.58	10,745.74	5574.59
Coagulation†	–	25,264.79	13,130.77
Microbiology‡	–	169.44	1279.36
Blood gas analysis	–	12,287.42	6606.97
D dimer	–	8620.25	4255.46
C-reactive protein	–	7481.50	3861.00
Ferritin	–	5758.36	3173.56
Procalcitonin	–	21,370.62	12,017.19
Troponin	–	12,613.64	6835.14
Interleukin 6		327.00	1918.40
ICU tests	–	43,775.00	0.00
*Total test costs*	66,931.38	177,359.34	81,490.33
***Drugs***			
Antivirals	–	10,034.86	691.36
Corticosteroids	–	1007.80	192.20
Tocilizumab	–	27,212.75	0.00
Others, anti-inflammatory	–	5115.51	53.44
Low-molecular-weight heparins	–	6272.06	1123.29
Antibiotics§	–	2868.16	1795.92
Mucolytics	–	383.74	81.40
ACE inhibitors/ARBs		64.10	18.99
Beta-blockers	–	29.21	8.95
Calcium antagonists	–	19.88	4.38
Alpha-blockers	–	19.08	6.84
Diuretics	–	7.92	1.74
Statins	–	19.76	5.37
Proton pump inhibitors	–	285.22	87.36
Analgesics	–	219.10	44.15
Antithrombotics	–	0	195.52
Drugs in ICU	–	30,519.30	0.00
*Total drug costs*	0.00	84,078.45	4310.91
***Imaging tests***			
Chest X-ray, portable	36,533.58	73,108.77	26,880.06
Computed tomography angiography	0.00	53,095.50	84,952.80
*Total imaging costs*	36,533.58	126,204.27	111,832.86
***Hospitalization***			
Medical visits	–	5,933,775.37	1951,26
Nursing hours	–	6,095,260.01	2004,36
ER stays	166,492,90	–	–
Hospitalization stays	–	1,570,847.58	350,648.36
Medical visits, ICU	–	103,660.58	0.00
Nursing hours, ICU	–	255,555.95	0.00
ICU stays§§	–	464,198.60	0.00
*Total hospitalization costs*	166,492,90	14,423,298.09	354,648.36
***TOTAL***			
TOTAL cost	269,957.86	14,810,940.15	552,282.36
Cost / patient	307.47	58,310.79	1709.85

ACE, angiotensin-converting enzyme; ARBs, angiotensin II receptor blockers; ICU, intensive care unit; RT-PCR, real-time polymerase chain reaction; SARS-CoV-2, severe acute respiratory syndrome coronavirus 2

* Emergency room blood tests include complete blood count, biochemical profile, and coagulation test. Drugs are included as general care costs in the ER

** Includes glycemia, cholesterol, triglycerides, potassium, sodium, albumin, total protein, GPT, GOT, GGT, CPK, LDH, calcium, magnesium, phosphate, transferrin, creatinine, urea, and bilirubin

† Includes: prothrombin time, APTT, and fibrinogen

‡ Includes blood culture, sputum culture, and tests for respiratory viruses (adenovirus, coronavirus, Middle Eastern Respiratory Syndrome, metapneumovirus, rhinovirus, enterovirus, influenza A, influenza B, parainfluenza, respiratory syncytial virus, *Bordetella pertussis, Chlamydophila pneumoniae*, *Mycoplasma pneumoniae*)

§ Includes amoxicillin/clavulanic acid, vancomycin, ceftriaxone, linezolid, levofloxacin, moxifloxacin, ciprofloxacin, piperacillin/tazobactam, imipenem, meropenem, ertapenem, daptomycin, and ceftoriden

§§ In the ICU, daily blood test and blood gas analysis

The main cost components in the SARS-CoV-2-positive cohort were hospital care and hospitalization, at €14,423,298 (97.4% of total); laboratory tests, at €177,359 (1.2%); imaging tests, at €126,204 (0.9%); and drugs, at €84,078 (0.6%).

ICU stays accounted for 5.3% of the total cost (€823,415.13). This cost corresponded to 9 patients (3.54% of the SARS-CoV-2-positive cohort) with a mean (SD) stay of 37.8 (12.9) days. ICU care accounted for 5.6% of the total cost for the overall SARS-CoV-2-positive cohort and 32.6% of the total cost for the SARS-CoV-2-positive cohort with ICU admission. ICU staffing costs were €103,661 (12.6%) for physicians and €255,556 (31.0%) for nursing staff. The cost of ICU stays for the SARS-CoV-2-positive cohort was €464,199 (56.4%).

The most used antiviral treatment was hydroxychloroquine combined with azithromycin, but lopinavir/ritonavir (€4335, 43.2%) and gamma interferon (€3594, 35.8%) were by far the largest cost components in this drug category. The most expensive treatment was tocilizumab (€27,213). Testing costs were mainly driven by RT-PCR tests (€94,418) and procalcitonin (€33,388). Imaging studies, at €238,037, accounted for just 1.5% of the total costs for all patients admitted (SARS-CoV-2-positive and -negative).

According to the results of the parametric sensitivity analysis, if the RT-PCR test-negative patients had not been treated, the total impact on the hospital’s budget would have been €14,693,256€ (€12,980 per patient). Likewise, if we assume a non-false-negative rate, the total cost would have been €19,376,071 (€17,117 per patient).

### Estimation of the National Level

In the conservative scenario, the total estimated impact of the COVID-19 pandemic on the Spanish public health budget was €9375 million; 1.5% of this cost corresponded to the ER cohort, 3.0% to the SARS-CoV-2-negative cohort, 63.2% to the SARS-CoV-2-positive cohort without ICU admission, and 32.3% to the SARS-CoV-2-positive cohort with ICU admission (Table 3). In the worst-case scenario, the total impact was €10,392 million (€139 million for the ER cohort, €284 million for the SARS-CoV-2-negative cohort, €5809 million for the SARS-CoV-2-positive cohort without ICU admission, and €4160 million for the SARS-CoV-2-positive cohort with ICU admission) (Table 3).

**Table 3:** Impact on Spanish public health budget

Cohort	No. of patients	Mean cost per patient	Budget impact (€ million)
***Conservative scenario***			
ER cohort	432,854	€307.47	€133.09
SARS-CoV-2-negative cohort	159,239	€1709.85	€272.28
SARS-CoV-2-positive cohort without ICU stay	113,558	€50,131.99	€5692.89
SARS-CoV-2-positive cohort with ICU stay	11,664	€280,955.81	€3277.07
**TOTAL**			**€9375.32**
***Worst-case scenario***			
ER cohort	451,730	€307.47	€138.89
SARS-CoV-2-negative cohort	166,183	€1709.85	€284.15
SARS-CoV-2-positive cohort without ICU stay	115,877	€50,131.99	€5809.14
SARS-CoV-2-positive cohort with ICU stay	14,806	€280,955.81	€4159.82
**TOTAL**			**€10,391.99**

At the time of writing (end of June 2020), the pandemic is still going on and the total number of cases, hospitalizations, and ICU admissions continues to rise. Assuming that the total number of hospitalizations remains below 185,000 and ICU admissions remain below 25,000, the estimated budget impact of the pandemic should remain below €15,000 million (Table 4).

**Table 4:** Impact on Spanish public health budget according to hospitalized and ICU cases

€ million	No. of ICU cases																			
No. of hospitalized cases	1500	3000	4500	6000	7500	9000	10,500	12,000	13,500	15,000	16,500	18,000	19,500	21,000	22,500	24,000	25,500	27,000	28,500	30,000
**5000**	613	959	1306	–	–	–	–	–	–	–	–	–	–	–	–	–	–	–	–	–
**20,000**	1414	1760	2106	2452	2799	3145	3491	3837	4184	4530	4876	5222	5568	–	–	–	–	–	–	–
**35,000**	2214	2560	2907	3253	3599	3945	4292	4638	4984	5330	5677	6023	6369	6715	7061	7408	7754	8100	8446	8793
**50,000**	3015	3361	3707	4053	4400	4746	5092	5438	5785	6131	6477	6823	7170	7516	7862	8208	8554	8901	9247	9593
**65,000**	3815	4161	4508	4854	5200	5546	5893	6239	6585	6931	7278	7624	7970	8316	8663	9009	9355	9701	10,047	10,394
**80,000**	4616	4962	5308	5654	6001	6347	6693	7039	7386	7732	8078	8424	8771	9117	9463	9809	10,156	10,502	10,848	11,194
**95,000**	5416	5763	6109	6455	6801	7147	7494	7840	8186	8532	8879	9225	9571	9917	10,264	10,610	10,956	11,302	11,649	11,995
**110,000**	6217	6563	6909	7256	7602	7948	8294	8640	8987	9333	9679	10,025	10,372	10,718	11,064	11,410	11,757	12,103	12,449	12,795
**125,000**	7017	7364	7710	8056	8402	8749	9095	9441	9787	10,134	10,480	10,826	11,172	11,518	11,865	12,211	12,557	12,903	13,250	13,596
**140,000**	7818	8164	8510	8857	9203	9549	9895	10,242	10,588	10,934	11,280	11,627	11,973	12,319	12,665	13,011	13,358	13,704	14,050	14,396
**155,000**	8618	8965	9311	9657	10,003	10,350	10,696	11,042	11,388	11,735	12,081	12,427	12,773	13,120	13,466	13,812	14,158	14,504	14,851	15,197
**170,000**	9419	9765	10,111	10,458	10,804	11,150	11,496	11,843	12,189	12,535	12,881	13,228	13,574	13,920	14,266	14,613	14,959	15,305	15,651	15,997
**185,000**	10,220	10,566	10,912	11,258	11,604	11,951	12,297	12,643	12,989	13,336	13,682	14,028	14,374	14,721	15,067	15,413	15,759	16,106	16,452	16,798
**200,000**	11,020	11,366	11,713	12,059	12,405	12,751	13,097	13,444	13,790	14,136	14,482	14,829	15,175	15,521	15,867	16,214	16,560	16,906	17,252	17,599
**215,000**	11,821	12,167	12,513	12,859	13,206	13,552	13,898	14,244	14,590	14,937	15,283	15,629	15,975	16,322	16,668	17,014	17,360	17,707	18,053	18,399
**230,000**	12,621	12,967	13,314	13,660	14,006	14,352	14,699	15,045	15,391	15,737	16,083	16,430	16,776	17,122	17,468	17,815	18,161	18,507	18,853	19,200
**245,000**	13,422	13,768	14,114	14,460	14,807	15,153	15,499	15,845	16,192	16,538	16,884	17,230	17,577	17,923	18,269	18,615	18,961	19,308	19,654	20,000
**260,000**	14,222	14,568	14,915	15,261	15,607	15,953	16,300	16,646	16,992	17,338	17,685	18,031	18,377	18,723	19,070	19,416	19,762	20,108	20,454	20,801
**275,000**	15,023	15,369	15,715	16,061	16,408	16,754	17,100	17,446	17,793	18,139	18,485	18,831	19,178	19,524	19,870	20,216	20,563	20,909	21,255	21,601
**290,000**	15,823	16,170	16,516	16,862	17,208	17,554	17,901	18,247	18,593	18,939	19,286	19,632	19,978	20,324	20,671	21,017	21,363	21,709	22,056	22,402
**305,000**	16,624	16,970	17,316	17,663	18,009	18,355	18,701	19,047	19,394	19,740	20,086	20,432	20,779	21,125	21,471	21,817	22,164	22,510	22,856	23,202

### Sensitivity analysis

The main sources of cost variation were physician salaries (range, €10,131 to €11,357/patient), nursing staff salaries (€10,274 to €11,215/patient), and general stays (€10,612 to €10,876/patient). The budget impact ranged from €14,741,399 to €16,524,924 for hospitals and from €15,050,529 (€10,344/patient) to €16,913,800 (€11,625/patient) for regions.

**Fig. 1:**
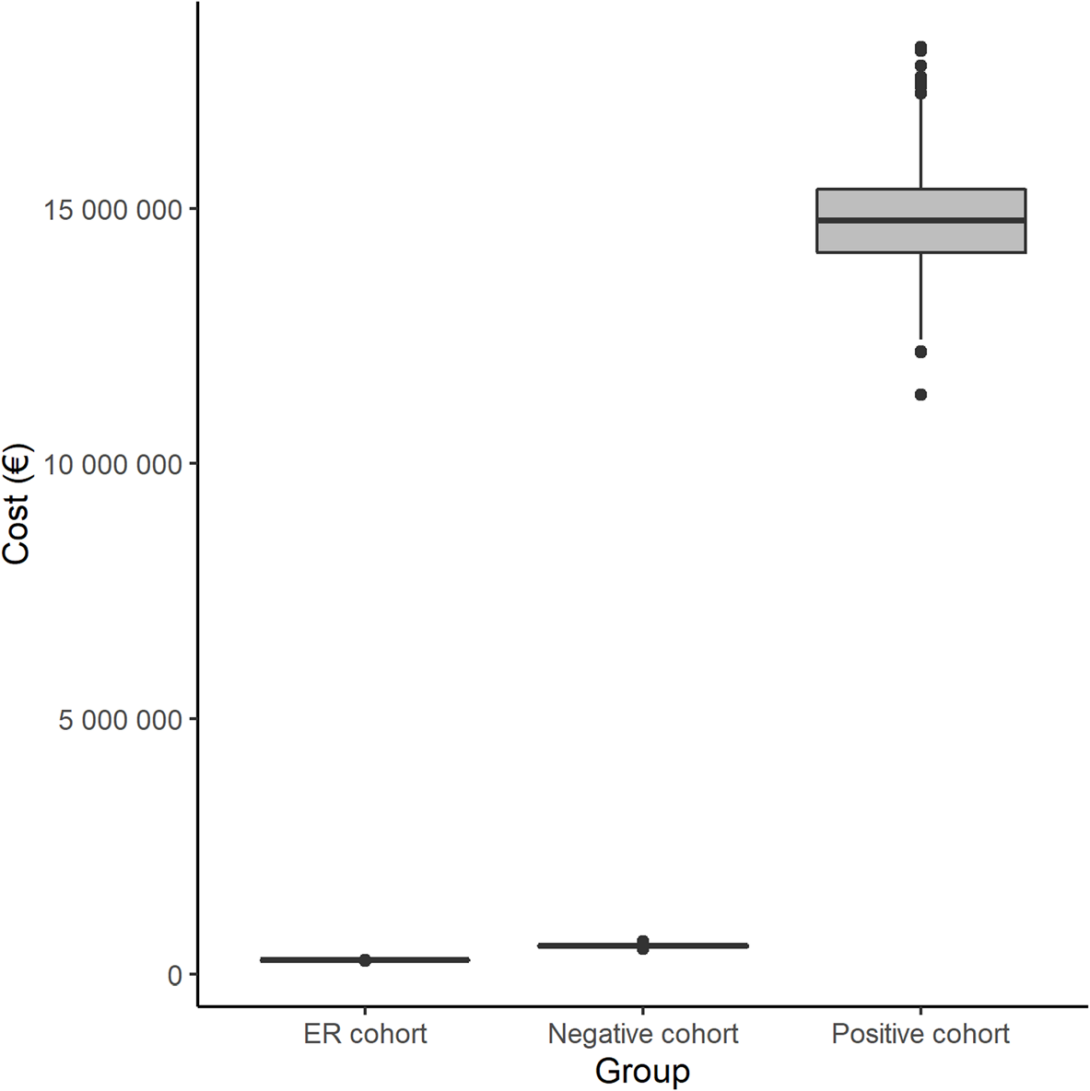
Sensitivity analysis of the economic impact overall and by cohort (bootstrapping method). SARS-CoV-2, severe acute respiratory syndrome coronavirus 2

The probabilistic analysis showed that the total median impact of the COVID-19 pandemic on the hospital budget was €15,581,235 (Fig. 1). By cohort, the median impact was €14,754,694 for the SARS-CoV-2-positive cohort, €551,592 for the SARS-CoV-2-negative cohort, and €269,947 for the ER cohort.

Median per-patients costs in the SARS-CoV-2-positive cohort were €53,373 for general stays, €3108 for ICU stays, €692 for laboratory tests, €497 for diagnostic tests, and €330 for drugs. In the SARS-CoV-2-negative cohort, the median per-patient costs were €1098 for general stays, €346 for diagnostic tests, €252 for blood tests, and €13 for drugs. The median cost per patient in the ER cohort was €307.

**Fig. 2:**
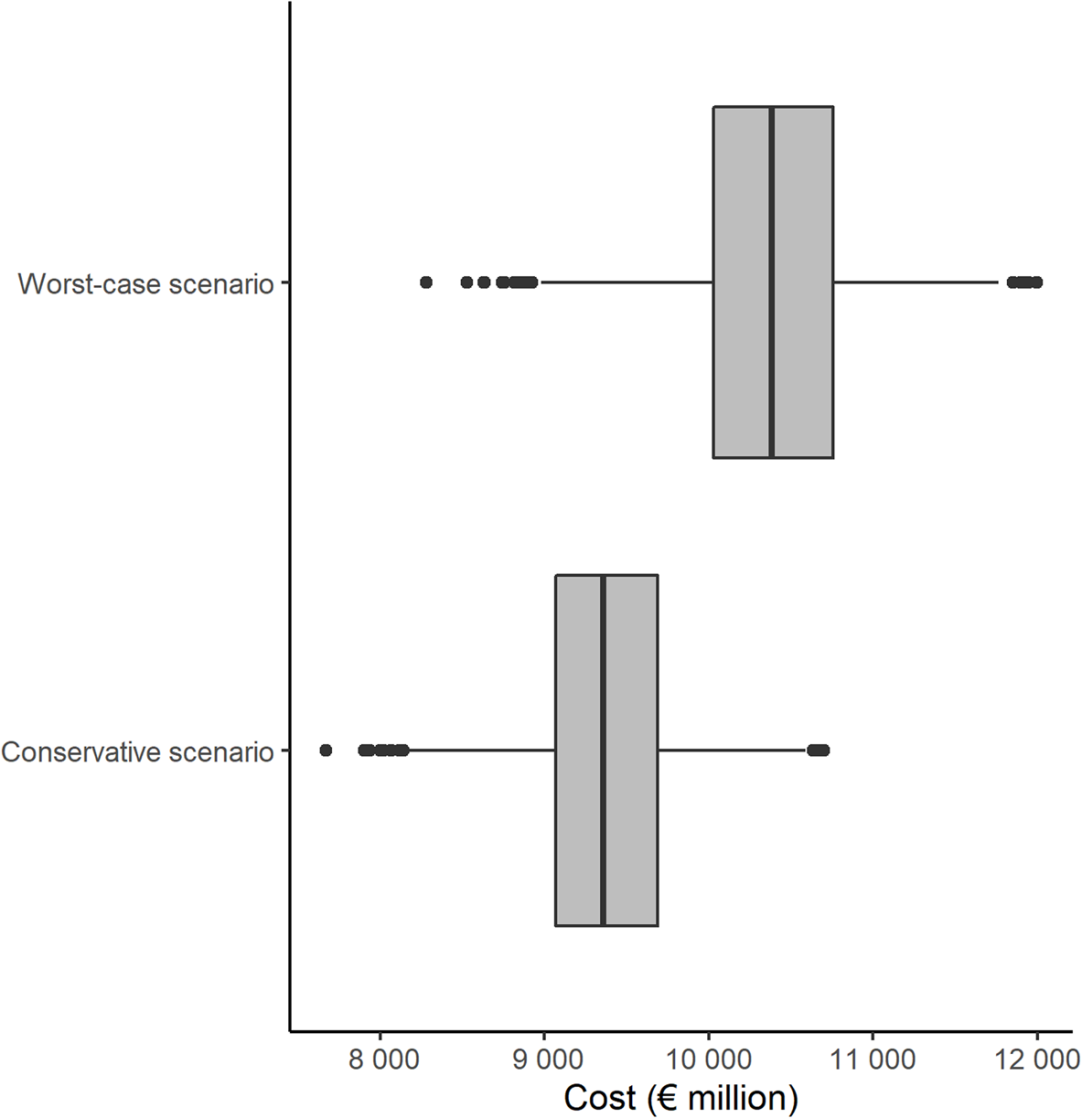
Impact of COVID-19 pandemic on the Spanish public health budget (bootstrapping method)

The median estimated impact on the Spanish public health budget was €9357 million (interquartile range (IQR), €9071 to €9689 million) for the conservative scenario and €10,385 million (IQR, €10,030 to €10,758 million) for the worst-case scenario (Fig. 2).

## Discussion

The COVID-19 impact on the Spanish public health budget during the peak of the pandemic (86 days), considering direct medical costs only, was estimated at over €9.4 billion (12.3% of total public health expenditure) [10]. This is greater than the impact reported for numerous conditions in Spain, such as multiple sclerosis (€1.4 billion) [11], cancer (€4.8 billion) [12], and diabetes (€5.8 billion) [13]. These figures give a picture of how big the cost of the pandemic has been at its peak in Spain. The overall impact on the healthcare system, however, can be assumed to be even greater, as care provision for other diseases was disrupted during the peak of the pandemic, as occurred in 68% of countries in Europe [14, 15]. In Spain, for example, the number of percutaneous coronary interventions to treat myocardial infarction fell by 40% during the pandemic [16], increasing future risks and potential costs.

The mean cost estimated for treating a patient with suspected or confirmed COVID-19 at our hospital was €10,744. While this is lower than the costs of preterm birth, specialized surgical procedures, or treatments for solid cancer, it is higher than those of most procedures in a medium-sized hospital such as ours, where treating a patient with septicemia requiring mechanical ventilation for more than 96 h, for example, costs €9087.

Our budget impact analysis of the COVID-19 pandemic in Spain will be a useful tool for hospital and department planning and preparedness purposes. Our findings may also be of help to other countries wishing to forecast the impact of the pandemic on their healthcare systems, although this would require adaptation to local procedures and costs. Cost-estimation studies are also needed to document the investment and use of public funds during the pandemic. Our estimates could also help healthcare authorities and governments design mitigation plans to protect the healthcare system and prevent staff burnout. Disease prevention is increasingly crucial for ensuring the well-being of both society and the economy.

Although our hospital was equipped with additional ICU beds during the initial phase of the pandemic, these were insufficient to meet all our mechanical ventilation needs, meaning that some patients needed to be transferred to other hospitals. We therefore performed a sensitivity analysis in which we varied the percentage of patients admitted to the ICU based on data from other published cohorts (10.2%) [8]. The results showed an increase in cost per patient from €10,744 to €13,411. ICU stays accounted for 5.6% of total costs, even though just 3.5% of patients required ICU care. The main drivers of costs were staff salaries and general and ICU stays (97.8%). Drug treatments accounted for just 0.6% of total costs and can therefore be considered a relatively small cost component.

Using a prediction model for prolonged hospital stays among patients with COVID-19 in China, Hong et al. [17] estimated a mean cost per patient of €925 (IQR, €636 to €1395), which is 11.6 times lower than the figure calculated in our study. This difference could be due to the relatively small sample size analyzed by Hong et al. and the exclusion of patients with severe disease. In addition, the study was not a formal cost-analysis study. Another recent Chinese study of 70 patients hospitalized for a median of 16 days (IQR, 10–20 days) estimated a cost of $6827 per episode of COVID-19 [18], which is closer to our figure. Nonetheless, the median length of stay for SARS-CoV-2-positive patients in our series was just 8 days (IQR, 5–15 days) but the cost per patient was much higher, at €50,132. This difference can largely be attributed to staff costs, as our study was performed during the peak of the pandemic, when the hospital was overstretched. Other possible reasons include cultural differences and differences in healthcare system organization and costs. A US study that developed a Monte Carlo simulation model based on the assumption that 80% of the population would become infected calculated a total median direct medical cost of $654 billion (95% CI: 615.8–692.8) [19], with a median cost of $14,366 (95% CI: 13,545-15,129) per hospitalized patient and $215 million (95% CI: 209–221) for symptomatic patients. Our study, however, is based on real-world data and is not comparable.

To date, two Spanish studies have been published. Rodríguez-Gonzalez et al. [20] performed a cost analysis in a referral hospital (*n* = 1255) with global costs (€ 0.44 million per 1000 hospitalized patient and € 408 per patient) similar to our results (€ 307 per patient). These differences may be due to the higher incidence in Madrid during the first wave of COVID-19. However, we also present an estimate of the budgetary impact on the national health system of € 9357 million, by carrying out a probabilistic sensitivity analysis that took into account different incidence scenarios of the disease. In the second study [21] they make a totally theoretical estimate based on gross domestic product and not on real data like us.

Retrospective cohort studies are prone to selection bias. In an attempt to minimize this risk, we included all patients with suspected SARS-CoV-2 infection who visited the ER at our hospital. One notable strength of our study is the use of individual-level data for both diagnostic tests and treatments.

Another limitation of our study is related to possible false-negative misclassifications. False-negative rates ranging from 16 to 66% have been reported for RT-PCR, although these have improved over time [7, 22]. The prevalence of SARS-CoV-2 infection also varies by region, although the mean age and sex of hospitalized patients in our cohort were similar to those reported at the national level (66 years and 55% males for general-stay patients and 63 years and 55% males for ICU patients) [8]. The main limitation of our study, however, is that our calculations are based on data from a single hospital and cannot therefore be generalized to hospitals with other characteristics. Furthermore, to calculate the national costs, we assumed a constant ratio between negative and positive cohorts. Although, we analyzed the uncertainty of this parameter in the sensitivity analysis, readers must take attention that this relationship might not be linear.

Finally, we did not analyze indirect costs, such as productivity loss, but as most of the patients in the cohort were elderly and healthcare provider perspective.

## Conclusions

The total estimated impact of COVID-19 on our hospital’s budget for a period of 86 days during the peak of the pandemic was €15.6 million, or €10,744 per patient. On extrapolating these estimates to Spain as a whole, the total direct medical cost accrued up to the end of June 2020 is €10.4 billion.

## Supplementary Information

**Additional file 1: Table S1.** Unit cost for seven autonomous communities.

## Data Availability

The data that support the findings of this study are available on request from the corresponding author.

## Abbreviations

COVID-19: Coronavirus disease 2019
SARS-CoV-2: Severe acute respiratory syndrome coronavirus 2
AEMPS: Spanish Agency of Medicines and Medical Products
ER: hospital’s emergency room
RT-PCR: real-time polymerase chain reaction
ICU: Intensive Care Unit
€: Euros
PPE: Personal protective equipment
IQR: interquartile range
