# Supplementary material for: Hospitalization budget impact during the COVID-19 pandemic in Spain
Source: Health Econ Rev. 2021 Nov 3;11:43. doi: 10.1186/s13561-021-00340-0 (PMC8565649; doi:10.1186/s13561-021-00340-0)
Supplement: Supplementary file 1 — Additional file 1: Table S1. Unit cost for seven autonomous communities. [file 13561_2021_340_MOESM1_ESM.docx]

**SUPPLEMENT MATERIAL**

Table S1 Unit cost for seven autonomous communities

Table S1 Unit cost for seven autonomous communities

|  | VALENCIAN COMMUNITY ^a^ | COMMUNITY OF MADRID ^b^ | NAVARRE ^c^ | BASQUE COUNTRY ^d^ | CATALONIA ^e^ | CANARY ISLANDS ^f^ | CANTABRIA ^g^ | ANDALUSIA ^h^ |
| --- | --- | --- | --- | --- | --- | --- | --- | --- |
| MEDICAL COST |  |  |  |  |  |  |  |  |
| Hospitalization (day) | 581,58 | 581,58 | 663,15 | 581,58 | 581,58 | 669,61 | 581,58 | 495,59 |
| Intensive Care unit (day) | 1365,29 | 1365,29 | 1490,68 | 1365,29 | 1105 | 2515,86 | 214 | 1365,29 |
| Emergency room | 310,17 | 175 | 413,76 | 174 | 110 | 334,82 | 165 | 110,49 |
| Emergency consultant | 189,49 | 90 | 204 | 78 | 60 | 227,78 | 69 | 189,49 |
| STAFF COST |  |  |  |  |  |  |  |  |
| Doctor (Day) | 121,95 | 121,95 | 121,95 | 121,95 | 121,95 | 121,95 | 121,95 | 121,95 |
| Nurse (Day) | 83,52 | 83,52 | 83,52 | 83,52 | 83,52 | 83,52 | 83,52 | 83,52 |
| TEST |  |  |  |  |  |  |  |  |
| Chest x-ray | 41,61 | 41,61 | 23,33 | 22 | 9 | 154,28 | 41,61 | 9,23 |
| Pulmonary Angiography | 235,98 | 235,98 | 940,89 | 448 | 1400 | 322,01 | 90 | 138,45 |
| **Analytics** |  |  |  |  |  |  |  |  |
| Albumin | 0,38 | 0,38 | 0,38 | 2 | 2,15 | 0,59 | 0,38 | 0,23 |
| Protein total | 0,5 | 0,5 | 0,5 | 2 | 2,15 | 0,59 | 0,5 | 0,7 |
| Urine proteins | 4,26 | 4,26 | 4,26 | 2 | 27,69 | 1,59 | 4,26 | 4,26 |
| GPT | 0,62 | 0,62 | 0,62 | 2 | 2,15 | 0,59 | 0,62 | 0,23 |
| GOT | 0,62 | 0,62 | 0,62 | 2 | 2,15 | 0,59 | 0,62 | 0,23 |
| Calcium | 0,63 | 0,63 | 0,63 | 2 | 2,45 | 0,59 | 0,63 | 0,47 |
| Magnesium | 0,63 | 0,63 | 0,63 | 2 | 2,45 | 0,59 | 0,63 | 0,47 |
| Phosphate | 0,62 | 0,62 | 0,62 | 2 | 3,07 | 0,59 | 0,62 | 0,7 |
| Chloride | 1,11 | 1,11 | 1,11 | 2 | 2,15 | 0,59 | 1,11 | 0,47 |
| Cholesterol | 0,59 | 0,59 | 0,59 | 2 | 2,04 | 1,16 | 0,59 | 0,7 |
| CK | 1,68 | 1,68 | 1,68 | 4 | 7,36 | 0,59 | 1,68 | 1,4 |
| CK-MB | 17,61 | 17,61 | 17,61 | 6 | 6,74 | 14,79 | 17,61 | 2,33 |
| Creatinine | 0,34 | 0,34 | 0,34 | 1 | 1,23 | 0,59 | 0,34 | 0,47 |
| ACL Creatinica | 0,17 | 0,17 | 0,17 | 2 | 0,17 | 0,17 | 0,17 | 0,17 |
| GGT | 0,79 | 0,79 | 0,79 | 2 | 3,07 | 0,59 | 0,79 | 0,23 |
| Glucose | 0,56 | 0,56 | 0,56 | 1 | 1,23 | 0,59 | 0,56 | 0,47 |
| HbA1c | 12,42 | 12,42 | 12,42 | 10 | 6,74 | 12,42 | 12,42 | 12,42 |
| LDH | 0,71 | 0,71 | 0,71 | 3 | 12,26 | 0,59 | 0,71 | 0,47 |
| K | 1,11 | 1,11 | 1,11 | 2 | 2,15 | 0,59 | 1,11 | 0,93 |
| Na | 1,11 | 1,11 | 1,11 | 2 | 2,15 | 0,59 | 1,11 | 0,93 |
| Triglycerides | 1,02 | 1,02 | 1,02 | 2 | 3,43 | 1,16 | 1,02 | 0,47 |
| Urea | 0,71 | 0,71 | 0,71 | 2 | 1,78 | 0,59 | 0,71 | 0,7 |
| Bilirubin | 0,6 | 0,6 | 0,6 | 2 | 1,23 | 0,59 | 0,6 | 0,47 |
| Blood gas | 11,03 | 11,03 | 11,03 | 5 | 11,03 | 43,74 | 11,03 | 3,49 |
| Ferritin | 7,18 | 7,18 | 7,18 | 10 | 6,74 | 14,79 | 7,18 | 9,95 |
| IL6 | 21,8 | 21,8 | 21,8 | 21,8 | 21,8 | 14,79 | 21,8 | 21,8 |
| Transferrin | 8,39 | 8,39 | 8,39 | 10 | 11,47 | 14,79 | 8,39 | 6,3 |
| Troponin | 16,51 | 16,51 | 16,51 | 16 | 12,26 | 14,79 | 16,51 | 11,65 |
| PCR | 6,5 | 40 | 116,6 | 10 | 86,46 | 14,79 | 6,5 | 7,94 |
| Procalcitonin | 30,27 | 30,27 | 30,27 | 30,27 | 21,36 | 14,79 | 30,27 | 30,27 |
| **Microbiology** |  |  |  |  |  |  |  |  |
| RT_PCR COVID test | 64,67 | 64,67 | 64,67 | 102 | 64,67 | 85,15 | 64,67 | 116,49 |
| Blood culture | 14,12 | 14,12 | 14,12 | 24 | 17,2 | 10,96 | 14,12 | 19,99 |
| Bacterial blood culture | 15,63 | 15,63 | 15,63 | 15,63 | 32,33 | 15,63 | 15,63 | 19,99 |
| Sputum | 14,12 | 14,12 | 14,12 | 14,12 | 14,12 | 12,25 | 14,12 | 10,17 |
| Sputum culture | 10,71 | 10,71 | 10,71 | 26 | 10,71 | 10,71 | 10,71 | 10,71 |
| Legionella Urine | 15,63 | 15,63 | 15,63 | 30 | 24,36 | 12,25 | 15,63 | 15,63 |
| Pneumonia Urine | 15,63 | 15,63 | 15,63 | 30 | 21,36 | 12,25 | 15,63 | 15,63 |
| Multi-test | 93,2 | 93,2 | 93,2 | 93,2 | 93,2 | 85,15 | 93,2 | 93,2 |
| Blood count | 3,26 | 3,26 | 3,26 | 4 | 3,26 | 3,24 | 3,26 | 5,3 |
| Group S | 9,75 | 9,75 | 9,75 | 4 | 9,75 | 9,75 | 9,75 | 6,62 |
| **Coagulation** |  |  |  |  |  |  |  |  |
| DimeroD | 8,41 | 8,41 | 8,41 | 16 | 12,29 | 12,29 | 8,41 | 7,94 |
| APTT | 8,41 | 8,41 | 8,41 | 4 | 8,41 | 12,29 | 8,41 | 4,77 |
| Tprotombo | 8,41 | 8,41 | 8,41 | 3 | 8,41 | 12,29 | 8,41 | 4,77 |
| Fibrinogen | 8,41 | 8,41 | 8,41 | 7 | 9,2 | 12,29 | 8,41 | 5,3 |
| FiO2 | 3,48 | 3,48 | 3,48 | 3,48 | 3,48 | 3,48 | 3,48 | 3,48 |
| Intensive Care Unit analytics (calculated) | 128,75 | 162,25 | 238,85 | 165,27 | 242,95 | 144,82 | 128,75 | 118,15 |
| Emergency room analytics (calculated) | 43,06 | 76,56 | 153,16 | 41 | 143,04 | 91,07 | 43,06 | 30,23 |

1. Decreto legislativo 1/2005, de 25 de febrero, por el que se aprueba el texto refundido de la ley de tasas de la Generalitat Valenciana (Ejercicio 2020).
2. Boletín Oficial de la Comunidad Autónoma de Madrid, BOCM núm. 198. Lunes, 21 de agosto de 2017. ORDEN 727/2017, de 7 de agosto, del consejero de Sanidad, por la que se fijan los precios públicos por la prestación de los servicios y actividades de naturaleza sanitaria de la red de centros de la Comunidad de Madrid.
3. RESOLUCIÓN 1564/2018, de 20 de diciembre, del Director Gerente del Servicio Navarro de Salud-Osasunbidea, por la que se establecen las tarifas por los servicios prestados por el Servicio Navarro de Salud-Osasunbidea., - Boletín Oficial de Navarra, de 22-01-2019
4. Osakidetza. Tarifas para facturación de servicios sanitarios y docentes de Osakidetza para el año 2020.
5. Diari Oicial de la Generalitat de Catalunya. SLT/30/2013, de 20 de febrero, por la que se aprueban los precios públicos del Servicio Catalán de la Salud.Núm. 6323 CVE-DOGC-B-13051092-2013
6. Boletín Oficial de Canarias. Servicio Canario de la Salud- Resolución de 29 de marzo de 2017, del Director, por la que se modifica la cuantía de los precios públicos de servicios sanitarios previstos en el Decreto 81/2009, de 16 de junio, por el que se establecen los precios públicos de los servicios sanitarios prestados por el Servicio Canario de la Salud y se fijan sus cuantías. núm. 67 boc-a-2017-067-1616
7. Boletín Oficial de Cantabria número 129, de 5 de julio de 2019, de la Orden SAN/35/2017, por la que se fijan las cuantías de los precios públicos de los servicios sanitarios prestados por el Servicio Cántabro de Salud.
8. Boletín Oficial de la Junta de Andalucía. Orden de 18 de noviembre de 2015, por la que se modifica la Orden de 14 de octubre de 2005, por la que se fijan los precios públicos de los servicios sanitarios prestados por centros dependientes del Sistema Sanitario Público de Andalucía. Núm. 228 página 15
